# Corneal nerve fiber loss relates to cognitive impairment in patients with Parkinson’s disease

**DOI:** 10.1038/s41531-021-00225-3

**Published:** 2021-09-09

**Authors:** Ning-Ning Che, Qiu-Huan Jiang, Guan-Xiao Ding, Si-Yuan Chen, Zhen-Xiang Zhao, Xue Li, Rayaz A. Malik, Jian-Jun Ma, Hong-Qi Yang

**Affiliations:** 1 Department of Neurology, Henan Provincial People’s Hospital, School of Clinical Medicine, Henan University, Zhengzhou, China; 2 Department of Neurology, Affiliated BenQ Hospital of Nanjing Medical University, Nanjing, China; 3 Nursing department, Henan Provincial People’s Hospital, Zhengzhou, China; 4 Department of Medicine, Weill Cornell Medicine-Qatar, Doha, Qatar; 5 Division of Cardiovascular Sciences, School of Medical Sciences, University of Manchester, Manchester, UK; 6 Department of Neurology, People’s Hospital of Zhengzhou University, School of Clinical Medicine, Zhengzhou University, Zhengzhou, China

**Keywords:** Parkinson's disease, Diagnostic markers

## Abstract

Cognitive impairment in Parkinson’s disease (PD) adversely influences quality of life. There is currently no available biomarker to predict cognitive decline in PD. Corneal confocal microscopy (CCM) has been used as a non-invasive tool for quantifying small nerve damage in PD. The present study investigated whether corneal nerve measures were associated with cognitive function in PD. Patients with PD were classified into those with normal cognitive function (PD-CN), mild cognitive impairment (PD-MCI), and dementia (PDD). Corneal nerve fiber density (CNFD), corneal nerve branch density (CNBD), and corneal nerve fiber length (CNFL) were quantified with CCM and compared with a control group. Sixty-five PD patients and thirty controls were studied. CNFD was decreased and CNBD was increased in PD patients compared to controls (*P* < 0.05). CNBD and CNBD/CNFD ratio was higher in PD-CN compared to controls. CNFD was positively correlated with the Montreal cognitive assessment (MoCA) score (*r* = 0.683, *P* < 0.001), but negatively associated with unified Parkinson disease rating scale (UPDRS)-part III (*r* = −0.481, *P* < 0.001) and total UPDRS scores (*r* = −0.401, *P* = 0.001) in PD patients. There was no correlation between CNFD and Levodopa equivalent daily dose (LEDD) (*r* = 0.176, *P* = 0.161). CNFD, CNBD, CNFL, and CNBD/CNFD ratio was lower with increasing Hoehn and Yahr stage. PD patients show evidence of corneal nerve loss compared with controls and corneal nerve parameters are associated with the severity of cognitive and motor dysfunction in PD. CCM could serve as an objective in vivo ophthalmic imaging technique to assess neurodegeneration in PD.

## Introduction

Parkinson’s disease (PD) is the second most common neurodegenerative disorder affecting the elderly worldwide^1^. Although the primary focus is on motor symptoms in PD, cognitive impairment, hyposmia, and autonomic dysfunction, referred to as non-motor symptoms (NMS), are increasingly recognized^2^. With the advance of disease course, patients will experience not only deteriorated motor function, they will also suffer much more from NMS, like cognitive impairment, hallucinations, psychosis symptoms, and eventually dementia etc. Over 40% of patients with normal cognition developed MCI within 6 years of being diagnosed with PD^3^ and 80% ultimately develop dementia^4^. Cognitive impairment is one of the most common NMS, and has an adverse effect on activity of daily life, care-giver burden, and quality of life^5^. Cognitive decline in PD encompasses the full spectrum of cognitive impairment include early mild subjective cognitive decline (SCD), mild cognitive impairment (PD-MCI), and Parkinson’s disease dementia (PDD)^6^. In SCD, cognitive impairment may be noted by the patient, family members or clinician, but cognitive test performance is normal^6^. In PD-MCI, insidious cognitive decline may be noticed by the patient, relative or clinician, but it does not interfere significantly with daily life^6,7^. While in PDD, cognitive decline can severely impair daily life and functional independence^8^. PD patients have a much higher risk of dementia as compared with the general population and the majority of patients with PD will develop some degree of cognitive impairment^3,9^. At present, there are no reliable biomarkers for predicting patients with increased risk of cognitive decline.

PD is traditionally regarded as a central neurodegenerative disorder, but peripheral nerve degeneration has been observed^9–11^. The overall incidence of peripheral neuropathy in PD patients is 19 to 55%^12^ and it may be associated with worse cognitive decline in PD patients^13^. Skin biopsy has confirmed the deposition of phosphorylated α-synuclein in and loss of the cutaneous nerve fibers^11^. Additionally, three studies utilizing corneal confocal microscopy (CCM) in patients with PD have shown corneal nerve loss which correlated with the severity of autonomic dysfunction and motor deficits^9,10,14^. More recently two studies have also shown a progressive loss of corneal nerve fibers in subjects with MCI and dementia using CCM^15,16^. Lim et al. have recently demonstrated a reduction in corneal nerve parameters, but no correlation with the duration, severity or subtype of PD and cognitive function^17^. Whether patients with PD-MCI and PDD have corneal nerve fiber loss is still unknown and is the focus of present study. We have quantified corneal nerve morphology in PD patients with different stages of cognitive decline and assessed the association of corneal nerve fiber loss with cognitive and motor function. We have also explored whether corneal nerve fiber parameters can predict cognitive decline in PD.

## Results

### Clinical and demographic profiles

A total of 75 PD patients were evaluated of whom 65 were included in the study. Among the 10 patients excluded, 7 had impaired glucose tolerance and 3 had corneal disease. The demographic and clinical profiles of PD patients (44.62% male; age 64.60 ± 6.95 years; age at onset 59.97 ± 6.84 years; disease duration 4.63 ± 2.53 years) and controls (53.33% male; age 62.43 ± 6.16 years) are summarized in Table 1. There were no significant differences in age, gender, education levels, BMI, plasma cholesterol, VitB_12_, and homocysteine concentrations between control and PD patients. As shown in Table 1, the MoCA scores were lower in PD patients than in the control group [24.00 (21.00, 28.00) vs 28.50 (27.00, 29.25), *P* < 0.0001].

**Table 1:** Demographic characteristics of participants.

	Controls (*n* = 30)	PD (*n* = 65)	PD-CN (*n* = 27)	PD-MCI (*n* = 23)	PDD (*n* = 15)
Age, mean ± SD, years	62.43 ± 6.16	64.60 ± 6.95	62.85 ± 6.97	65.48 ± 5.95	66.40 ± 8.04
Sex (M/F)	16/14	29/36	12/15	11/12	7/8
Education, mean ± SD, years	7.93 ± 3.85	8.26 ± 3.95	9.70 ± 3.01	7.09 ± 4.34	7.47 ± 4.24
BMI, mean ± SD, kg/m^2^	23.74 ± 3.19	22.92 ± 2.42	22.98 ± 2.16	22.80 ± 2.34	22.47 ± 2.31
Chol, mean ± SD, mmol/L	4.06 ± 1.04	3.99 ± 0.65	3.95 ± 0.71	3.93 ± 0.61	4.14 ± 0.64
Vit B12, mean ± SD, pg/ml	253.40 ± 93.89	257.41 ± 99.18	250.23 ± 92.89	252.09 ± 112.59	278.00 ± 91.29
Hcy, mean ± SD, umol/L	12.39 ± 2.41	11.78 ± 2.42	11.63 ± 2.22	11.55 ± 2.34	12.42 ± 2.91
Disease duration, mean ± SD, years	NA	4.63 ± 2.53	3.29 ± 1.44	4.65 ± 2.80 c*	7.00 ± 1.89 d***
Hoehn and Yahr Stage	NA	2.00 (1.00,3.00)	2.00 (1.00,2.00) b*	3.00 (2.00,4.00)	4.00 (3.00,4.00) d***
UPDRS-I, mean ± SD	NA	13.51 ± 7.23	11.37 ± 5.39	13.17 ± 6.87	15.87 ± 6.51
UPDRS-II, mean ± SD	NA	16.59 ± 7.69	13.22 ± 5.50	16.26 ± 8.78 c**	23.13 ± 5.07 d***
UPDRS-III, mean ± SD	NA	35.43 ± 14.18	26.41 ± 10.71 b**	39.00 ± 13.01	46.20 ± 11.75 d***
Total UPDRS, mean ± SD	NA	65.52 ± 23.15	51.00 ± 16.27 b*	68.44 ± 23.80 c*	85.20 ± 16.24 d***
LEDD (mg)	NA	500.00 (406.25,643.75)	500.00 (375.00,575.00)	573.92 ± 181.26	544.17 ± 164.61
SCOPA-AUT, mean ± SD	NA	15.74 ± 6.75	12.48 ± 5.67	15.43 ± 5.99 c**	22.07 ± 5.33 d***
Olfactory (olfactory dysfunction, %)	NA	73.85	55.56	82.61	93.33 d**
HAMA-14, mean ± SD	NA	11.94 ± 5.63	15.70 ± 6.56	16.22 ± 6.97	16.53 ± 5.33
HADA-24, mean ± SD	NA	16.08 ± 6.36	12.07 ± 6.23	11.09 ± 5.67	13.00 ± 4.47
PDQ-39, mean ± SD	NA	41.12 ± 14.66	32.52 ± 10.79 b*	41.74 ± 13.15 c**	55.67 ± 11.16 d***
MoCA, score	28.50 (27.00, 29.25)	24.00 (21.00, 28.00) a***	28.00(27.00, 29.00) b***	23.00(22.00, 24.00) c**	13.40 ± 3.83 d***
CNFD, mean ± SD, no./mm^2^	34.33 ± 3.78	25.83 ± 4.73***	29.26 ± 3.21 b***	25.04 ± 2.67 c**	20.86 ± 4.67 d***
CNBD, mean ± SD, no./mm^2^	24.58 ± 8.23	31.77 ± 14.15 a*	39.54 ± 13.67 b*	31.20 ± 10.22 c**	18.62 ± 10.12 d***
CNFL, mean ± SD, mm/mm^2^	15.86 ± 2.27	14.43 ± 3.31 a*	16.40 ± 2.53 b**	13.91 ± 1.81	11.69 ± 4.13 d***
CNBD/CNFD	0.72 ± 0.24	1.20 ± 0.44 a***	1.34 ± 0.38	1.26 ± 0.44	0.69 (0.58,1.33) d**

Numbers are expressed as mean ± SD or median (interquartile range). For variables following a normal distribution, two groups were compared using independent samples *t*-test and multiple comparisons were performed using analysis of variance (ANOVA) with Bonferroni as post hoc test. For variables following a non-normal distribution or non-homoscedasticity, two groups were compared with non-parametric Mann–Whitney test and multiple comparisons were performed using non-parametric Kruskal–Wallis test. Categorical variables were compared with Chi-square tests and Fisher’s exact tests. Abbreviations: *NA* not available, *PD-CN* Parkinson’s disease with normal cognitive function, *PD-MCI* Parkinson’s disease with mild cognitive impairment, *PDD* Parkinson’s disease dementia, *UPDRS* unified Parkinson’s disease rating scale, *LEDD* levodopa equivalent daily dose, *Hcy* Homocysteine, *SCOPA-AUT* the scale for outcomes in PD for autonomic symptoms, *HAMA-14* the 14-item Hamilton anxiety rating scale, *HADA-24* the 24-item Hamilton depression rating scale, *PDQ-39* the 39-item Parkinson’s disease questionnaire, *MoCA* Montreal cognitive assessment, *CNFD* corneal nerve fiber density, *CNBD* corneal nerve branch density, *CNFL* corneal nerve fiber length (*P < 0.05, **P < 0.01, ***P < 0.001).

^a^Significant difference between Controls and PD.

^b^Significant difference between PD-CN and P-MCI.

^c^Significant difference between PD-MCI and PDD.

^d^Significant differences between PD-CN and PDD.

### CCM measurement between PD and control group

**Fig. 1:**
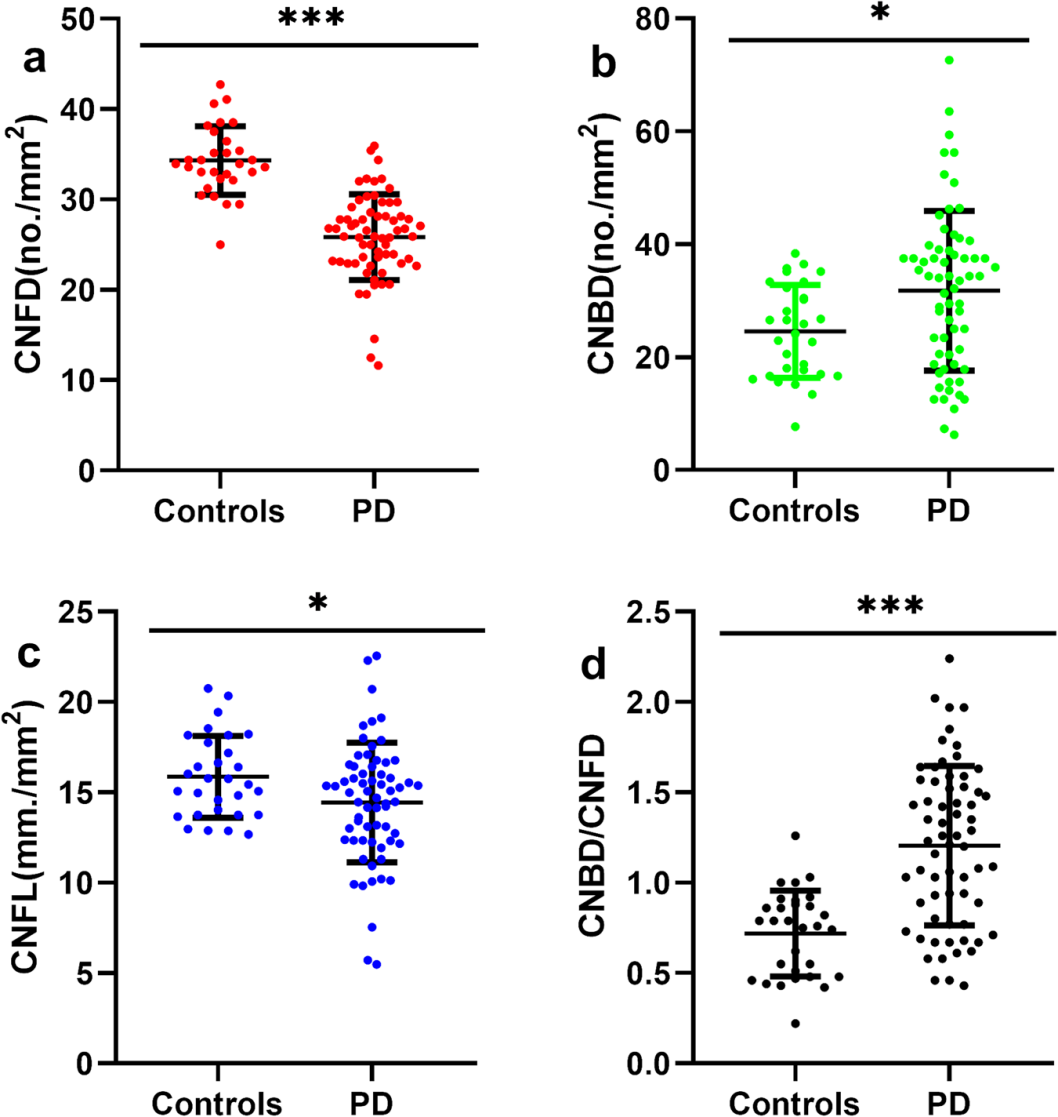
Corneal nerve fiber parameters in controls and patients with PD. CNFD and CNFL were decreased (**a**, **c**) and CNBD and CNBD/CNFD ratio were increased (**b**, **d**) in PD as compared to the control group with independent samples *t*-test. Errors bars represent mean ± standard deviation (*P < 0.05, **P < 0.01, ***P < 0.001). Abbreviations: PD Parkinson’s disease, CNFD corneal nerve fiber density, CNBD corneal nerve branch density, CNFL corneal nerve fiber length.

CNFD was significantly lower in PD patients compared to controls (no./mm^2^, 25.83 ± 4.73 vs 34.33 ± 3.78; mean difference, 8.49; 95% CI, 6.55 to 10.42; *P* = 0.000) (Fig. 1). CNBD was higher in PD patients compared to controls (no./mm^2^, 31.77 ± 14.15 vs 24.58 ± 8.23; mean difference, −7.18; 95% CI, −12.71 to −1.66; *P* = 0.011). CNFL was significantly lower in PD patients to controls (mm/mm^2^, 14.43 ± 3.31 vs 15.86 ± 2.27; mean difference,1.43; 95% CI, 0.10 to 2.76; *P* = 0.035). The CNBD/CNFD ratio was significantly higher in PD compared to controls (1.20 ± 0.44 vs 0.72 ± 0.24; mean difference, −0.49; 95% CI, −0.66 to −0.31; *P* = 0.000).

### CCM measurement between PD with different degrees of cognitive impairment

**Fig. 2:**
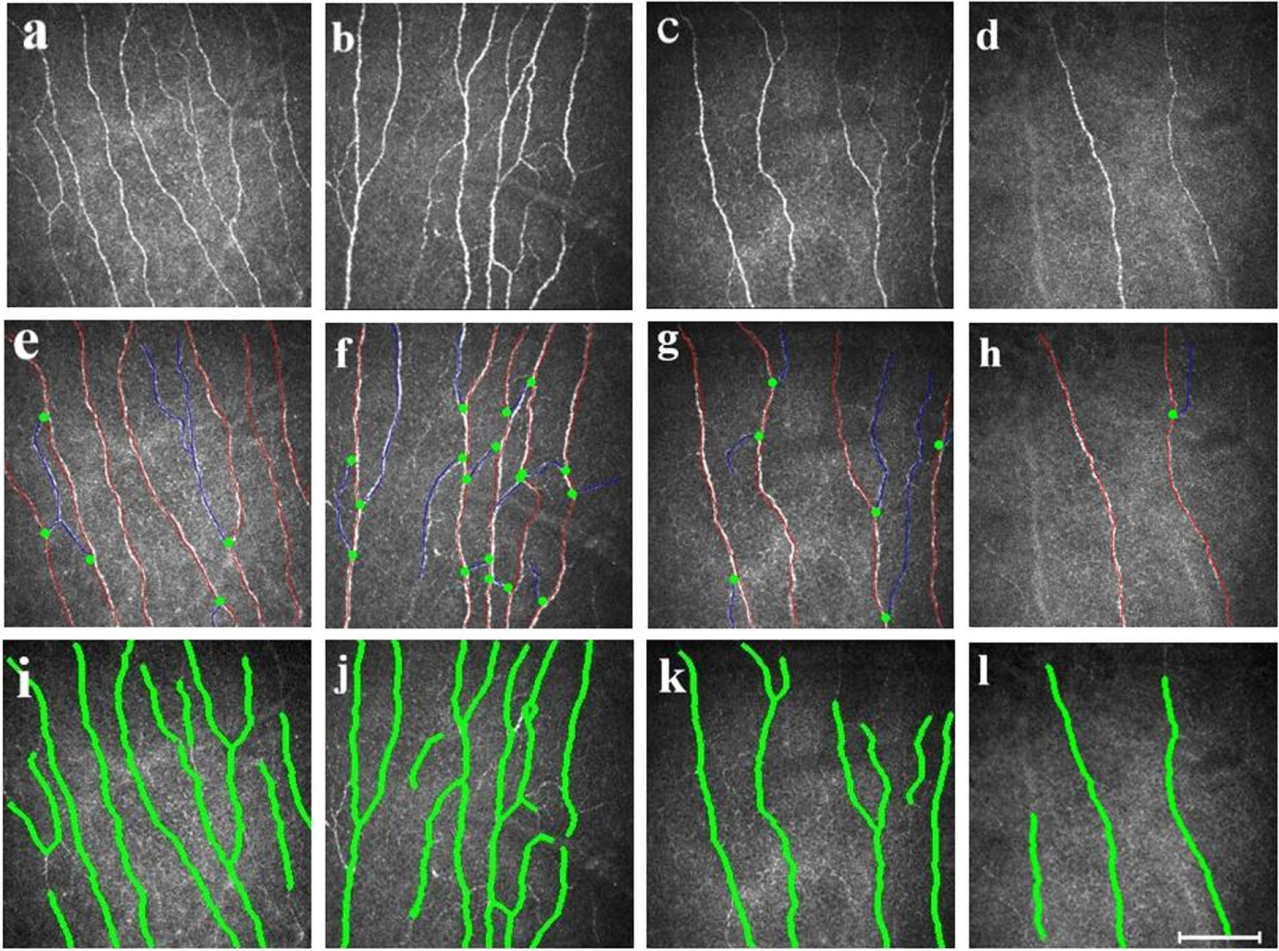
Representative CCM images in healthy controls, PD-CN, PD-MCI, and PDD. The corneal nerve plexus is beaded, linear homogeneous and highly reflective (**a**–**d**). Nerve fiber trunks are highlighted in red; branch origins are represented by the green dots (**e**–**h**) and corneal nerve fiber length is shown in green line (**i**–**l**). Images **e**–**h** were analyzed with the manual software (CCMetrics) and images **i**–**l** were marked with the automated version (ACCMetrics). PD-CN patients showed decreased CNFD and increased CNBD compared to the control group. PD-MCI and PDD patients showed a progressive reduction in CNFD, CNBD, and CNFL. Abbreviations: CCM corneal confocal microscopy, PD-CN Parkinson’s disease with cognitively normal, PD-MCI Parkinson’s disease with mild cognitive impairment, PDD Parkinson’s disease dementia, CNFD corneal nerve fiber density, CNBD corneal nerve branch density, CNFL corneal nerve fiber length. Scale bar = 100 um.

**Fig. 3:**
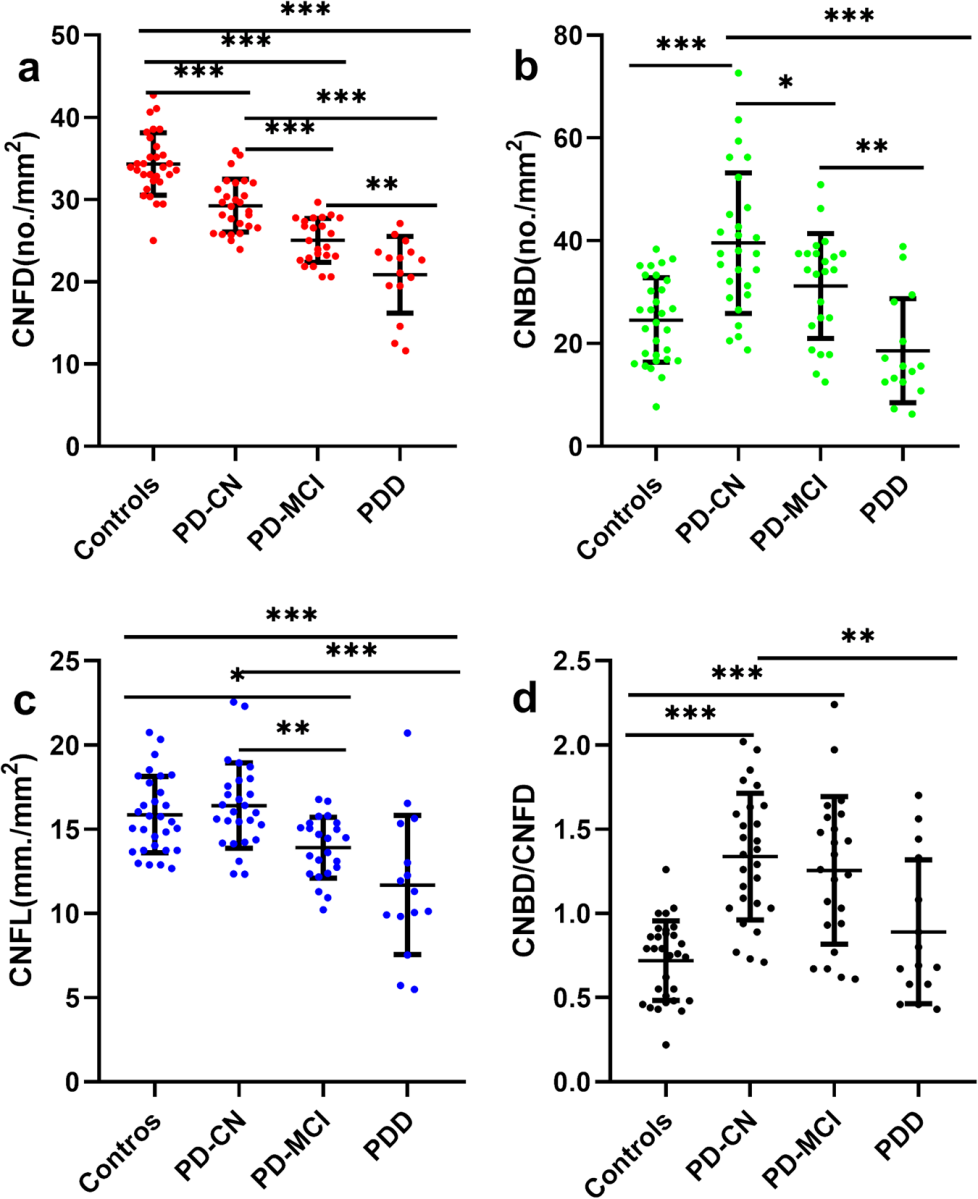
CCM parameters in healthy controls and PD patients with a varying degree of cognitive dysfunction. CNFD, CNBD, CNFL, and CNBD/CNFD ratio in PD-CN, PD-MCI, and PDD group were compared with that in the control group and dot plots were generated. CNFD decreased with cognitive decline (**a**). CNBD, CNFL and CNBD/CNFD radio was increased in PD-CN compared to controls with a trend for a decrease with cognitive impairment in PD (**b**–**d**). Multiple comparisons were performed using analysis of variance (ANOVA) with Bonferroni as post hoc test and Kruskal–Wallis test. Errors bars represent mean ± standard deviation (*P < 0.05, **P < 0.01, ***P < 0.001). Abbreviations: PD Parkinson’s disease, PD-CN Parkinson’s disease with cognitively normal, PD-MCI Parkinson’s disease with mild cognitive impairment, PDD Parkinson’s disease dementia, CNFD corneal nerve fiber density, CNBD corneal nerve branch density, CNFL corneal nerve fiber length.

To assess the relationship between corneal nerve morphology and severity of cognitive impairment, PD patients were classified into 3 subgroups: PD-CN [MoCA 28.00 (27.00–29.00), *n* = 27], PD-MCI [MoCA 23.00 (22.00–24.00), *n* = 23], and PDD (MoCA 13.40 ± 3.83, *n* = 15). The representative morphology of the corneal nerve fibers in each subgroup is shown in Figs 2 and 3. CNFD was lower in all three PD subgroups compared to controls (*P* < 0.001). CNFD in PD-MCI (no./mm^2^, 25.04 ± 2.67 vs 29.26 ± 3.21; mean difference, 4.22; 95% CI, 1.51 to 6.94; *P* = 0.000) and PDD (no./mm^2^, 20.86 ± 4.67 vs 29.26 ± 3.21; mean difference, 8.39; 95% CI, 5.32 to 11.48; *P* = 0.000) groups was significantly lower than those in patients with PD-CN. CNFD in the PDD group was also significantly lower than that in the PD-MCI group (no./mm^2^, 20.86 ± 4.67 vs 25.04 ± 2.67; mean difference, 4.17; 95% CI, 0.99 to 7.35; *P* = 0.004).

CNBD was significantly higher in patients with PD-CN compared with the control group (no./mm^2^, 39.54 ± 13.67 vs 24.58 ± 8.23; mean difference, 14.96; 95% CI, 7.26 to 22.67; *P* = 0.000). PDD patients had a significantly lower CNBD compared with PD-CN (no./mm^2^, 18.62 ± 10.12 vs 39.54 ± 13.67; mean difference, 20.92; 95% CI, 11.57 to 30.28; *P* = 0.000) and PD-MCI (no./mm^2^, 18.62 ± 10.12 vs 31.22 ± 10.22; mean difference, 3.57; 95%CI, 2.94 to 22.22; *P* = 0.004). CNBD in patients with PD-MCI was lower than that in the PD-CN group (*P* = 0.046).

CNFL was comparable between patients with PD-CN and the control group (mm/mm^2^, 16.40 ± 2.53 vs 15.86 ± 2.27; *P* = 1.000, by Kruskal–Wallis test). CNFL in patients with PD-MCI (mm/mm^2^, 13.91 ± 1.81 vs 16.40 ± 2.53, *P* = 0.007) and PDD (mm/mm^2^, 11.69 ± 4.13 vs 16.40 ± 2.53, *P* = 0.000) was significantly lower compared to that in PD-CN patients.

The CNBD/CNFD ratio was significantly higher in PD-CN (1.34 ± 0.38 vs 0.72 ± 0.24, *P* = 0.000) and PD-MCI group (1.26 ± 0.44 vs 0.72 ± 0.24, *P* = 0.000) compared to that in controls, whilst it was lower in PDD patients compared to that in patients with PD-CN (*P* = 0.006).

### Correlation of corneal nerve parameters with clinical and neurological outcomes

**Fig. 4:**
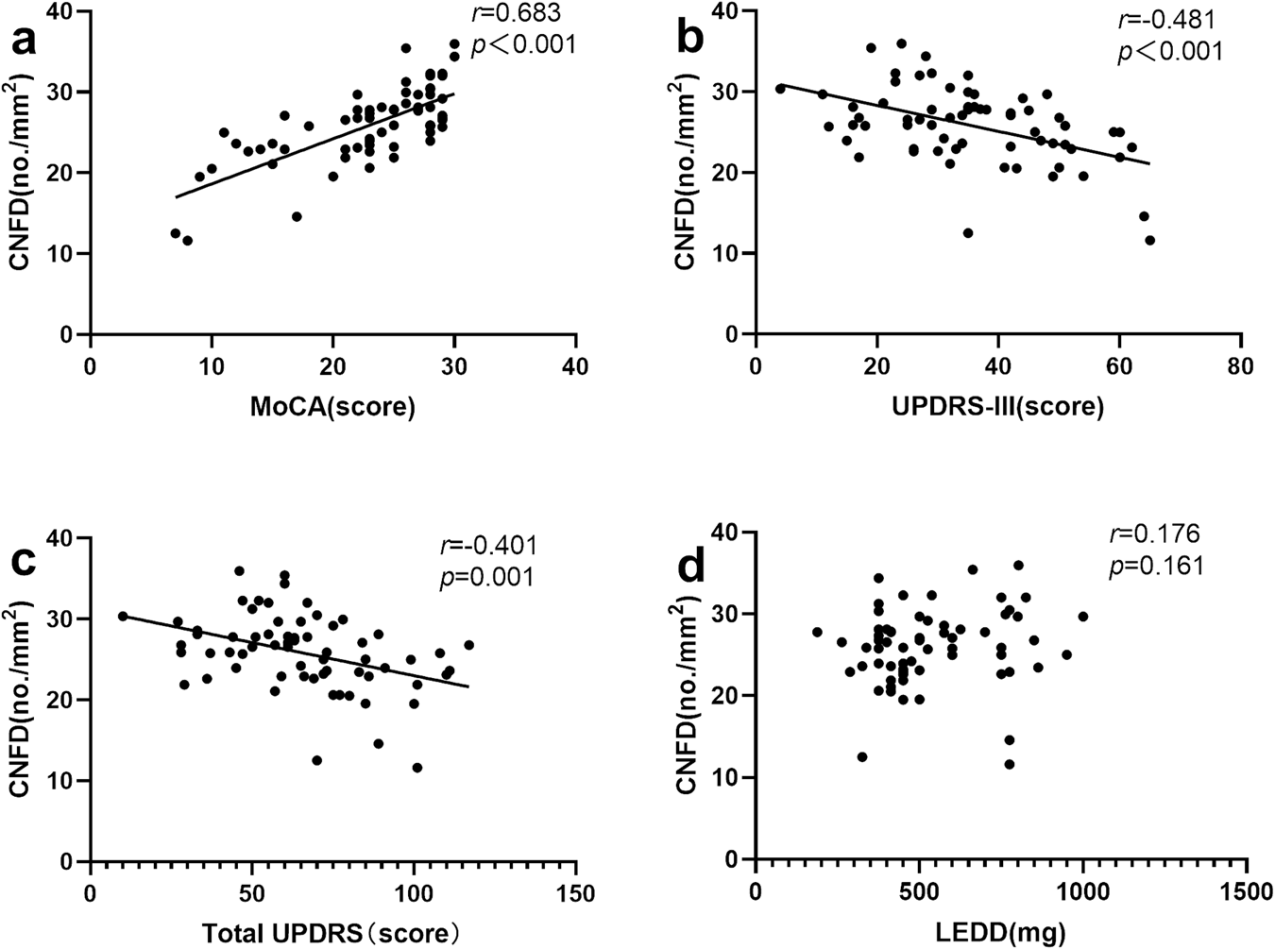
Association between CNFD with MoCA, part III and total UPDRS score and LEDD. The correlation was performed with Pearson (**a**, **d**) or Spearman (**b**, **c**) correlation analyses with standardized correlation coefficients Abbreviations: CNFD corneal nerve fiber density, MoCA Montreal cognitive assessment, UPDRS unified Parkinson’s disease rating scale, LEDD levodopa equivalent daily dose.

**Fig. 5:**
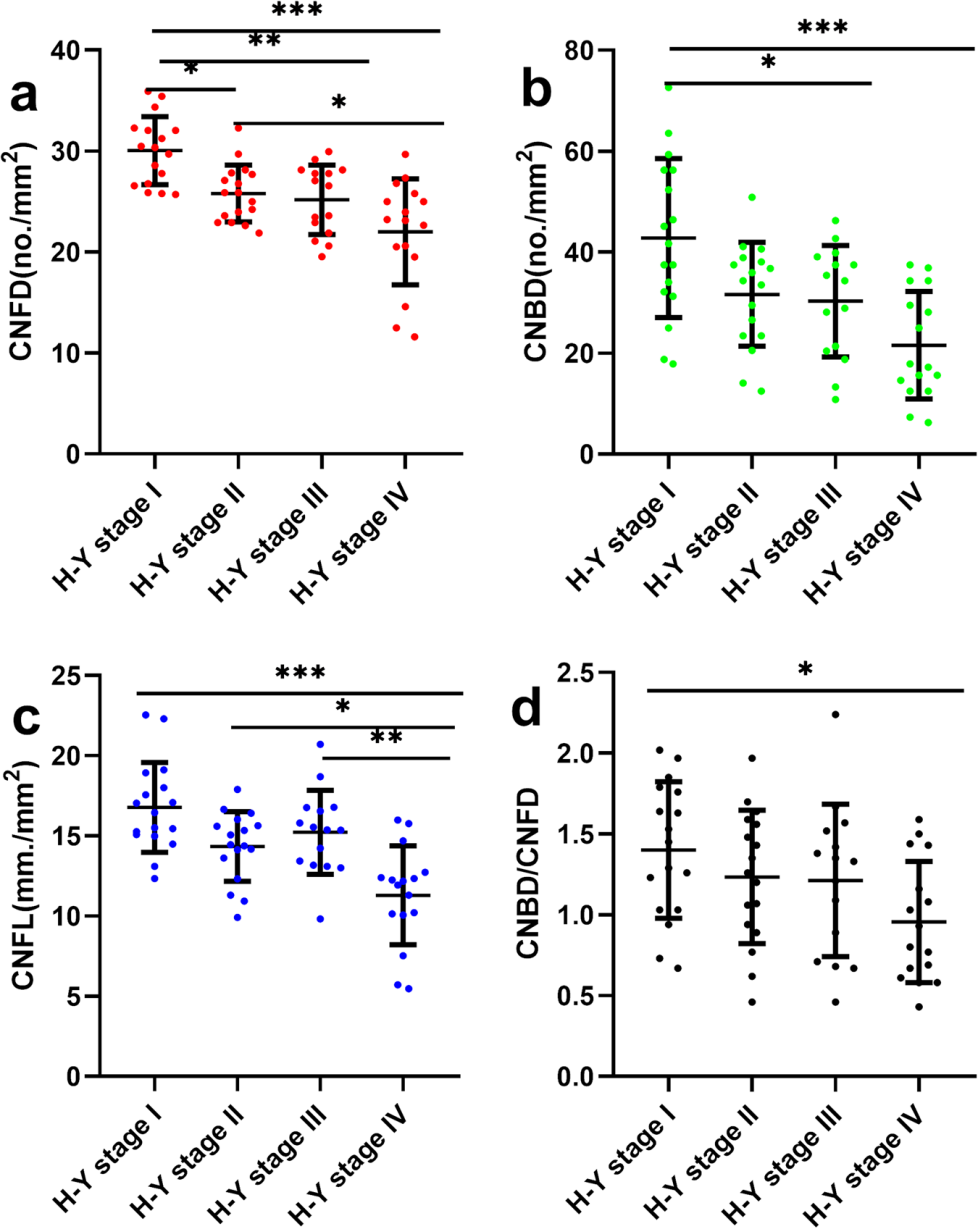
Association between CCM parameters and disease severity. CNFD (**a**), CNBD (**b**), CNFL (**c**), and CNBD/CNFD ratio (**d**) decreased progressively with increased H-Y stage. Multiple comparisons were performed using analysis of variance (ANOVA) with Bonferroni as post hoc test and Kruskal–Wallis test. Errors bars represent mean ± standard deviation (*P < 0.05, **P < 0.01, ***P < 0.001). Abbreviations: H-Y Hoehn-Yahr stage, PD-CN Parkinson’s disease with cognitively normal, PD-MCI Parkinson’s disease with mild cognitive impairment, PDD Parkinson’s disease dementia, CNFD corneal nerve fiber density, CNBD corneal nerve branch density, CNFL corneal nerve fiber length.

CNFD was positively associated with MoCA (Spearman’s correlation coefficient *r* = 0.683, *P* < 0.001), but negatively associated with UPDRS-III (Pearson’s correlation coefficient *r* = −0.481, *P* < 0.001) and total UPDRS scores (Pearson’s correlation coefficient *r* = −0.401, *P* = 0.001) in PD patients (Fig. 4). There was no correlation between CNFD and LEDD (Spearman’s correlation coefficient *r* = 0.176, *P* = 0.161). PD patients had lower CNFD, CNBD, CNFL, and CNBD/CNFD ratio with higher H-Y stage (Fig. 5).

### Correlation of cognitive function with clinical and neurological outcomes

To explore the factors affecting cognitive function, univariate analysis with MoCA as the dependent variable showed a significant association with CNFD (*b* = 0.910, *P* < 0.0001), CNBD (*b* = 0.248, *P* < 0.0001), CNFL (*b* = 1.021, *P* < 0.0001), disease duration (*b* = −1.316, *P* < 0.0001), H-Y stage (*b* = −2.760, *P* < 0.0001), UPDRS-III (*b* = −0.200, *P* < 0.0001), and SCOPA-AUT (*b* = −0.439, *P* < 0.0001). In multiple linear regression analysis, CNFD, CNBD, CNFL, disease duration, H-Y stage, UPDRS-III, and SCOPA-AUT were independent variables and stepwise selection was used to select risk variables. After adjusting for age and sex, MoCA was associated with CNFD (*b* = 0.678, *P* < 0.0001), disease duration (*b* = −0.713, *P* = 0.001), and SCOPA-AUT (*b* = −0.165, *P* = 0.036) (adjusted *R*^2^ = 0.615, *P* < 0.001) (Table 2).

**Table 2:** Multiple linear regression analysis for cognitive function in Parkinson’s disease.

	Coefficient	*SE*	*r*	*t*	*P* value
**MoCA**					
**CNFD, no./mm**^**2**^	0.678	0.112	0.531	6.050	0.000
**Disease duration**	−0.713	0.203	−0.298	−3.508	0.001
**SCOPA-AUT**	−0.165	0.077	−0.185	−2.142	0.036

In this model, MoCA was set as the dependent variable, with disease duration, H-Y, CNFD, CNBD, CNFL, UPDRS-III, and SCOPA-AUT as independent variables. Adjusted for age and gender, all the variables considered in the fitted model had *P* < 0.05, adjusted *R*^*2*^ = 0.615, *P* < 0.001. Abbreviations: *MoCA* Montreal cognitive assessment, *CNFD* corneal nerve fiber density, *SCOPA-AUT* the scale for outcomes in PD for autonomic symptoms, *H-Y* Hoehn and Yahr Stage, *CNBD* corneal nerve branch density, *CNFL* corneal nerve fiber length, *UPDRS* unified Parkinson’s disease rating scale.

### Diagnostic utility of CCM

**Fig. 6:**
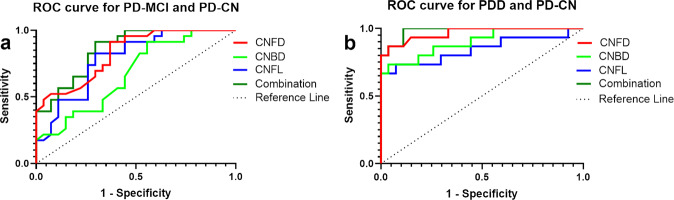
The ROC for CCM parameters to distinguish PD-MCI and PDD from PD-CN. The accuracy of ROC predicting PDD (**b**) was higher than PD-MCI (**a**). Abbreviations: ROC receiver operating characteristic, CNFD corneal nerve fiber density, CNBD corneal nerve branch density, CNFL corneal nerve fiber length, PD-MCI Parkinson’s disease with mild cognitive impairment, PDD Parkinson’s disease with dementia, PD-CN Parkinson’s disease with normal cognitively function, AUC area under the curve.

ROC analysis showed that CNFD, CNBD, and CNFL could discriminate between PD-MCI and PD-CN with an area under the curve (AUC) of 82.85% (95% CI, 71.82–93.88%), 67.47% (95% CI, 52.63–82.31%), and 78.74% (95% CI, 66.23–91.26%). Using a CNFD cutoff of <27.98 no./mm^2^, the sensitivity and specificity for PD-MCI was 91.30% and 62.96%. Using a CNBD cutoff of <40.23 no./mm^2^, the sensitivity and specificity for PD-MCI was 91.30% and 44.40%. Using a CNFL cutoff of <15.37 mm/mm^2^, the sensitivity and specificity for PD-MCI was 78.26 and 70.37%. Moreover, a combination of three parameters resulted in an increased AUC of 85.99% (95% CI, 76.09–95.89%), with sensitivity and specificity of 91.30% and 70.37% (Fig. 6a).

The AUC distinguishing PDD from PD-CN for CNFD, CNBD, and CNFL was 96.67% (95%CI, 91.62–100%), 90.12% (95%CI, 80.12–100%), 84.44% (95%CI, 69.87–99.02%), respectively. Using a CNFD cutoff of <25.00 no./mm^2^, the sensitivity and specificity for PDD was 86.67% and 96.30%. Using a CNBD cutoff of <20.47 no./mm^2^, the sensitivity and specificity for PDD was 73.33% and 96.30%. Using a CNFL cutoff of <12.29 mm/mm^2^, the sensitivity and specificity for PDD was 66.67 and 100%. Furthermore, a combination of three parameters resulted in an increased AUC of 98.27% (95% CI, 95.36–100%), the sensitivity and specificity was 100% and 88.89%, respectively (Fig. 6b).

## Discussion

The main finding of this study is that corneal nerve loss was associated with cognitive impairment and neurological disability in patients with PD. Indeed, clinicopathological studies indicate involvement of the peripheral nervous system in PD. Two kinds of peripheral neuropathy have been proposed in PD according to the peripheral nerves involved^18,19^. One, is a medium-large fiber neuropathy (Aα/β fibers involved), which is thought to be related to levodopa therapy and consequent vitamin B12 deficiency^12,20^. It usually develops in advanced PD patients^19^ and can be diagnosed with nerve conduction studies. This large fiber neuropathy may affect balance and gait, and increases the chance of falls^21^. A recent study has shown that the frequency of falls almost tripled in PD patients with neuropathy as compared to PD patients without neuropathy^22^. Ceravolo et al. have shown that longer time exposure to levodopa and increasing age are associated with the development of neuropathy^12^. The other type of neuropathy is characterized by a small fiber neuropathy (Aδ and C fibers involved) observed in the early stage of PD. It can be measured with a skin biopsy and/or CCM Podgorny^18,19^. Skin biopsy has confirmed deposition of phosphorylated α-synuclein in intraepidermal nerve fibers which correlated negatively with intraepidermal nerve fiber density (IENFD)^11^. Nolano et al. have found that IENFD loss is associated with disease duration and severity^23^. There was no significant difference in IENFD in PD patients who were levodopa-naive or on levodopa treatment^24^. Our data has also revealed no association between CNFD and LEDD, which is consistent with other studies^10,14^ indicating that small fiber neuropathy is not related to levodopa treatment.

The present study showed a lower CNFD and higher CNBD in patients with PD which is consistent with Kass-Iliyya et al.^14^, indicating concomitant nerve degeneration and regeneration^25^. We further show that CCM alterations correlate with cognitive status and CNFD was independently associated with cognitive function after adjusting for age and sex. It is speculated that in the early stage of cognitive decline in PD-CN, a reduction in CNFD may activate compensatory mechanism which lead to increased CNBD and CNBD/CNFD ratio. As the disease continues to advance, nerve regeneration may be impaired, which can result in the decline of cognitive function. Indeed, the patients with PD-CN had relative mild PD with a disease duration of 3.29 years and H-Y score of 2, compared to PD-MCI and PDD and had an increased CNBD/CNFD ratio compared to the other groups. However, in the PDD group, disease duration was 7.0 years and the H-Y score was 4 indicative of greater loss of both peripheral and central dopaminergic neurones^26^. Recently, Lim et al. have shown a decrease in all corneal nerve parameters, but no correlation with the MoCA score^17^. However, their cohort had a wide mixture of PD severity and minimal cognitive dysfunction. Alternatively, Misra et al. studied a small cohort of patients with more severe PD (Hoehn and Yahr grade 3/4) and showed a reduction in corneal nerve fiber density and correlation with cognitive function^9^. There are differences in the degree of corneal nerve loss between the current study and other published studies in patients with PD^9,10,14,17^, which may reflect different populations with differing severity of PD and cognitive dysfunction.

In the present study we show that CCM has good diagnostic utility for differentiating PD patients with mild cognitive impairment and dementia from those without cognitive impairment. Ponirakis et al. have shown reduced CNBD in MCI and dementia patients compared to controls^15^. These subjects with MCI may have differed from PD-MCI as they were recruited from the general population and the patients with dementia included those with Alzheimer’s disease, vascular dementia, and mixed dementia. Our PD patients all had an underlying synucleinopathy^27^. Patients with other neurodegenerative conditions like multiple sclerosis, Wilson’s disease, and Friedreich’s ataxia also showed a decreased CNBD compared to controls^28–30^. Thus, different etiologies may impact differently on corneal nerve fibers^31^.

Another important finding is that patients with PD-MCI and PDD have more serious autonomic symptoms compared to PD-CN patients and indeed multiple linear regression analyses showed that MoCA was associated with CNFD and SCOPA-AUT. Autonomic nerve fibers are thinly myelinated or unmyelinated nerve fibers^32^ and autonomic dysfunction may suggest small fiber neuropathy in PD. In a newly proposed clinical subtypes in PD, patients were divided into three subgroups: the mild motor predominant, intermediate, and diffuse malignant^33^. The last type involved not only severe motor symptoms, but also autonomic dysfunction and cognitive impairment and was associated with faster progression, worse prognosis, and shorter survival^34^. Association between small fiber impairment (represented by autonomic dysfunction and CCM) and cognitive decline may suggest that small fiber neuropathy may be a clinical sign of diffuse malignant subtype of PD.

Our study has some limitations. The small sample size limits generalization of this result to all PD patients. The cross-sectional design cannot address whether CCM parameters at baseline might be used to predict cognitive decline and a longitudinal follow-up study with a larger sample size would be required. The PD patients are also clinically diagnosed and did not undergo a more definitive DAT SPECT scan, although this cannot exclude atypical PD. Third, the clinical spectrum of cognitive impairment in PD includes SCD, PD-MCI, and PDD. The purpose of this study is to explore the corneal nerve functions through CCM in PD, thus SCD is not included in this study because the cognitive test is in normal range, making diagnosis difficult. Cognitive function was determined by the MoCA score, instead of comprehensive cognitive testing. This was a hospital-based study and patient selection bias was unavoidable. We are also aware that dry eye, which is more frequent in PD may influence corneal nerve morphology, although this was not found to have a major influence^35^. Finally, CCM is only available at tertiary hospitals and research facilities which currently limits more widespread use in PD clinics.

Nevertheless, for the first time, we show that CCM demonstrates an association between corneal nerve loss and cognitive function in PD. The underlying basis for this association is not clear but it may suggest a common underlying neurodegenerative process involving both the central and peripheral nervous system. The relatively good diagnostic outcomes to differentiate the different groups suggest that corneal nerve fiber might be a useful marker to differentiate patients with PD and differing degrees of cognitive impairment and neurological disability.

## Methods

### Participants inclusion criteria

Patients with PD were recruited from the department of Neurology, Henan Provincial People’s Hospital between March 2017 and October 2019. PD was diagnosed according to the 2015 MDS clinical diagnostic criteria for Parkinson’s disease^36^. Examinations of all cardinal manifestations were carried out with the MDS-unified Parkinson disease rating scale (UPDRS). Patients diagnosed with atypical parkinsonism (progressive supranuclear palsy, cortical basal ganglia degeneration, and multiple system atrophy), and secondary parkinsonism (drug-induced, immune-mediated, inflammatory, infectious, traumatic or neoplasm, etc.) were excluded from the study. Healthy controls were included from either volunteers or spouse of PD patients who had no history of movement disorder or cognitive impairment. Patients or healthy controls younger than 40 or older than 85 years of age were excluded from the study. Participants with a history of eye surgery, eye inflammation, glaucoma, corneal disease, thyroid eye disease were excluded. Other causes of peripheral neuropathy were excluded by undertaking a history of alcohol use and an assessment of vitamin B_12_ and folate, serum electrophoresis to exclude multiple myeloma, cryoglobulinemia, macroglobulinemia, and oral glucose tolerance test to exclude impaired glucose tolerance and diabetes. All subjects agreed to participate in the study and written informed consent was obtained. The clinical profiles of each participant were carefully reviewed by experienced neurologists who were specialized in movement disorders (J.-J. M. and H.-Q.Y.). The study was approved by the ethics committee of Henan Provincial People’s Hospital.

### Definitions of cognitive impairment

PD-cognitively normal (PD-CN) was defined as a clinical diagnosis of PD with no cognitive complaints and normal cognitive performance with a Montreal cognitive assessment (MoCA, Beijing Version) score ≥26 points.

PD-mild cognitive impairment (PD-MCI) was defined according to the Movement Disorder Society Diagnostic Criteria for Mild Cognitive Impairment in Parkinson’s Disease^37^. Here, MoCA cutoff score of <26 was used to diagnose PD-MCI (level I category).

PD-dementia (PDD) was defined according to the Clinical diagnostic criteria for dementia associated with Parkinson’s disease^38^. In this study, a clinical diagnosis of PD with deficits in at least two cognitive domains severe enough to affect daily life and normal functioning was diagnosed, with a MoCA cutoff score <21.

### Demographics

Age, gender, education levels, BMI, serum vitamin B_12_, homocysteine, and cholesterol were assessed in all subjects. Disease duration was defined as the time between presentation with first motor symptoms and the present study. Levodopa equivalent daily dose (LEDD) was assessed according to the levodopa conversion formula^39^. In brief, 100 mg levodopa = 133 mg entacapone = 1 mg pramipexole = 5 mg ropinirole = 10 mg selegiline = 1 mg rasagiline = 100 mg amantadine.

### Motor symptoms and non-motor symptom evaluation

Part I, II, and III sub-scales of the unified Parkinson’s disease rating scale (UPDRS) and Hoehn and Yahr staging (H-Y) were scored for all patients with PD^40^. Olfactory function was assessed with an olfactory kit for Parkinson’s disease (Jiangsu Parkinsense Biotech Co., Ltd, Nanjing, China). The olfactory kit consists of twelve smell cards, with four olfactory options each and the subject is required to choose the correct one. All subjects are required to complete all odor tests and the result is represented by the overall score. Olfactory dysfunction was identified by a score <8. Autonomic symptoms were evaluated by the scale for outcomes Parkinson’s disease for autonomic symptoms (SCOPA-AUT)^41^. These include symptoms of the gastrointestinal tract, urinary tract, cardiovascular system, thermoregulation, pupil activity, and sexual function with a higher score indicating more severely autonomic dysfunction. Quality of life was evaluated with the 39-item Parkinson’s disease questionnaire (PDQ-39) with a higher score indicating a poorer quality of life. The 14-item Hamilton anxiety rating scale (HAMA-14) and the 24-item Hamilton depression rating scale (HAMD-24) were used to assess anxiety and depression, respectively. The evaluation of motor and non-motor symptoms in PD patients were all performed in the “on” state.

### Corneal confocal microscopy

Images of the corneal sub-basal plexus were prepared using a Heidelberg Retina Tomograph III with a Rostock Cornea Module (HRT III RCM; Heidelberg Engineering GmbH, Heidelberg, Germany)^42^. Lidocaine was used to anesthetize each eye and the subject was seated comfortably and instructed to fixate on an outer fixation light. The CCD camera was used to correctly position the applanating cap onto the cornea. Images from the central cornea at the level of the sub-basal plexus were captured using the “section” mode by an experienced examiner according to an established protocol^43^. We chose 4–6 CCM images, the best quality of pictures for each eye in the center area of corneal, which were selected and analyzed using validated, semi-automated, purpose-written software (CCMetrics, Imaging Science and Biomedical Engineering, Manchester, UK) and the automated version (ACCMetrics)^44^. Three corneal nerve parameters were analyzed: (a) corneal nerve fiber density (CNFD): the number of all main nerve fibers per square millimeter; (b) corneal nerve branch density (CNBD): the number of branch nerves originating from the main nerve; and (c) corneal nerve fiber length (CNFL): the sum of length of all nerve fibers per square millimeter^45^. A decrease in CNFD represents a proximal loss of corneal nerve fibers and decreased or increased CNBD represents more distal nerve degeneration or regeneration, respectively. Thus CNBD/CNFD ratio was introduced here to represent the total ability of nerve regeneration.

### Statistical analysis

The Shapiro–Wilk test was used to check the data for normality. For variables following a normal distribution, numbers are expressed as mean ± standard deviation (SD). Two groups were compared using independent samples *t*-test and multiple comparisons were performed using analysis of variance (ANOVA) with Bonferroni as post hoc test. For variables following a non-normal distribution or non-homoscedasticity, numbers are expressed as median (interquartile range). Two groups were compared with non-parametric Mann–Whitney test and multiple comparisons were performed using non-parametric Kruskal–Wallis test. Categorical variables were compared with Chi-square tests and Fisher’s exact tests. The correlation between corneal nerve measures and clinical characteristics were explored with Pearson or Spearman correlation analyses, and the standardized correlation coefficients were presented. MoCA were set as the dependent variable. Age, gender, BMI, VitB_12_, disease duration, H-Y stage, UPDRS-I, UPDRS-II, UPDRS-III, LEDD, SCOPA-AUT, CNFD, CNBD, and CNFL were considered as independent variables in univariate linear regression analyses. The variables with *P* < 0.05 at the bivariate level were considered in multiple linear regression analyses. Stepwise selection was used to select variables. The receiver operating characteristic (ROC) curve was used to analyze the capability of CNFD, CNBD, and CNFL for distinguishing patients with PD-MCI from PD-CN, and PDD from PD-CN. All analyses were carried out using SPSS version 22.0 (IBM Corporation, Armonk, NY, USA). Dot plots and ROC curve were generated using GraphPad Prism version 8.0 (GraphPad Software, Inc, San Diego, CA, USA). *P* value of <0.05 was considered statistically significant.

### Reporting summary

Further information on research design is available in the Nature Research Reporting Summary linked to this article.

## Supplementary information

Reporting Summary

## Data Availability

The data that support the findings of this study are available from the corresponding authors upon reasonable request.

## References

[1] Morris R (2020). Cognitive function in people with and without freezing of gait in Parkinson’s disease. NPJ Parkinsons Dis..

[2] Che NN, Yang HQ (2020). Potential use of corneal confocal microscopy in the diagnosis of Parkinson’s disease associated neuropathy. Transl. Neurodegener..

[3] Pigott K (2015). Longitudinal study of normal cognition in Parkinson disease. Neurology.

[4] Hely MA, Reid WG, Adena MA, Halliday GM, Morris JG (2008). The Sydney multicenter study of Parkinson’s disease: the inevitability of dementia at 20 years. Mov. Disord..

[5] Tang Y (2020). Cognitive function and quality of life in Parkinson’s disease: a cross-sectional study. J. Parkinsons Dis..

[6] Galtier I, Nieto A, Lorenzo JN, Barroso J (2019). Subjective cognitive decline and progression to dementia in Parkinson’s disease: a long-term follow-up study. J. Neurol..

[7] Chen H (2020). The compensatory phenomenon of the functional connectome related to pathological biomarkers in individuals with subjective cognitive decline. Transl. Neurodegener..

[8] Hoogland J (2017). Mild cognitive impairment as a risk factor for Parkinson’s disease dementia. Mov. Disord..

[9] Misra SL, Kersten HM, Roxburgh RH, Danesh-Meyer HV, McGhee CN (2017). Corneal nerve microstructure in Parkinson’s disease. J. Clin. Neurosci..

[10] Podgorny PJ, Suchowersky O, Romanchuk KG, Feasby TE (2016). Evidence for small fiber neuropathy in early Parkinson’s disease. Parkinsonism Relat. Disord..

[11] Donadio V (2014). Skin nerve alpha-synuclein deposits: a biomarker for idiopathic Parkinson disease. Neurology.

[12] Ceravolo R (2013). Neuropathy and levodopa in Parkinson’s disease: evidence from a multicenter study. Mov. Disord..

[13] Merola A (2017). Peripheral neuropathy as marker of severe Parkinson’s disease phenotype. Mov. Disord..

[14] Kass-Iliyya L (2015). Small fiber neuropathy in Parkinson’s disease: a clinical, pathological and corneal confocal microscopy study. Parkinsonism Relat. Disord..

[15] Ponirakis G (2019). Association of corneal nerve fiber measures with cognitive function in dementia. Ann. Clin. Transl. Neurol..

[16] Al-Janahi E (2020). Corneal nerve and brain imaging in mild cognitive impairment and dementia. J. Alzheimers Dis..

[17] Lim SH (2020). Corneal confocal microscopy detects small fibre neurodegeneration in Parkinson’s disease using automated analysis. Sci. Rep..

[18] Cossu G, Melis M (2016). The peripheral nerve involvement in Parkinson disease: a multifaceted phenomenon. Parkinsonism Relat. Disord..

[19] Muller T (2013). Peripheral neuropathy in Parkinson’s disease: levodopa exposure and implications for duodenal delivery. Parkinsonism Relat. Disord..

[20] Jugel C (2013). Neuropathy in Parkinson’s disease patients with intestinal levodopa infusion versus oral drugs. PLoS One.

[21] Adewusi JK (2018). Peripheral neuropathic pain in idiopathic Parkinson’s disease: prevalence and impact on quality of life; a case controlled study. J. Neurol. Sci..

[22] Beaulieu ML, Muller M, Bohnen NI (2018). Peripheral neuropathy is associated with more frequent falls in Parkinson’s disease. Parkinsonism Relat. Disord..

[23] Nolano M (2018). Small fiber pathology parallels disease progression in Parkinson disease: a longitudinal study. Acta Neuropathol..

[24] Nolano M, Provitera V, Lanzillo B, Santoro L (2011). Neuropathy in idiopathic Parkinson disease: an iatrogenic problem?. Ann. Neurol..

[25] Nolano M (2008). Sensory deficit in Parkinson’s disease: evidence of a cutaneous denervation. Brain.

[26] Kordower JH (2013). Disease duration and the integrity of the nigrostriatal system in Parkinson’s disease. Brain.

[27] Yang K (2018). Cognitive characteristics in Chinese non-demented PD patients based on gender difference. Transl. Neurodegener..

[28] Petropoulos IN (2017). Corneal confocal microscopy: an imaging endpoint for axonal degeneration in multiple sclerosis. Invest Ophthalmol. Vis. Sci..

[29] Sturniolo GC (2015). Small fiber peripheral neuropathy in Wilson disease: an in vivo documentation by corneal confocal microscopy. Invest Ophthalmol. Vis. Sci..

[30] Pagovich OE (2018). Corneal confocal microscopy: neurologic disease biomarker in Friedreich ataxia. Ann. Neurol..

[31] Petropoulos IN (2020). The utility of corneal nerve fractal dimension analysis in peripheral neuropathies of different etiology. Transl. Vis. Sci. Technol..

[32] Vinik A, Ullal J, Parson HK, Casellini CM (2006). Diabetic neuropathies: clinical manifestations and current treatment options. Nat. Clin. Pr. Endocrinol. Metab..

[33] De Pablo-Fernandez E (2017). Association of autonomic dysfunction with disease progression and survival in Parkinson disease. JAMA Neurol..

[34] Armstrong MJ, Okun MS (2020). Diagnosis and treatment of Parkinson disease: a review. JAMA.

[35] Ferdousi M (2018). No relation between the severity of corneal nerve, epithelial, and keratocyte cell morphology with measures of dry eye disease in Type 1 diabetes. Invest Ophthalmol. Vis. Sci..

[36] Postuma RB (2015). MDS clinical diagnostic criteria for Parkinson’s disease. Mov. Disord..

[37] Litvan I (2012). Diagnostic criteria for mild cognitive impairment in Parkinson’s disease: movement disorder society task force guidelines. Mov. Disord..

[38] Emre M (2007). Clinical diagnostic criteria for dementia associated with Parkinson’s disease. Mov. Disord..

[39] Tomlinson CL (2010). Systematic review of levodopa dose equivalency reporting in Parkinson’s disease. Mov. Disord..

[40] Goetz CG (2008). Movement disorder society-sponsored revision of the unified Parkinson’s disease rating scale (MDS-UPDRS): scale presentation and clinimetric testing results. Mov. Disord..

[41] Visser M, Marinus J, Stiggelbout AM, Van Hilten JJ (2004). Assessment of autonomic dysfunction in Parkinson’s disease: The SCOPA-AUT. Mov. Disord..

[42] Dabbah MA, Graham J, Petropoulos IN, Tavakoli M, Malik RA (2011). Automatic analysis of diabetic peripheral neuropathy using multi-scale quantitative morphology of nerve fibres in corneal confocal microscopy imaging. Med. Image Anal..

[43] Tavakoli, M. & Malik, R. A. Corneal confocal microscopy: a novel non-invasive technique to quantify small fibre pathology in peripheral neuropathies. *J. Vis. Exp.* 2194 (2011). 10.3791/2194 PMC3182640 21248693

[44] Ferdousi. MTM, Morris. INPJ, Zhivov. NPA, Ziegler D (2015). Normative values for corneal nerve morphology assessed using corneal confocal microscopy: a multinational normative data set. Multicent. Study.

[45] Alam U (2017). Diagnostic utility of corneal confocal microscopy and intra epidermal nerve fibre density in diabetic neuropathy. PLoS One.

